# The Role of Norrish Type-I Chemistry in Photoactive Drugs: An *ab initio* Study of a Cyclopropenone-Enediyne Drug Precursor

**DOI:** 10.3389/fchem.2020.596590

**Published:** 2020-12-22

**Authors:** Spencer J. Léger, Barbara Marchetti, Michael N. R. Ashfold, Tolga N. V. Karsili

**Affiliations:** ^1^Department of Chemistry, University of Louisiana at Lafayette, Lafayette, LA, United States; ^2^Department of Chemical Engineering, University of Louisiana at Lafayette, Lafayette, LA, United States; ^3^School of Chemistry, University of Bristol, Bristol, United Kingdom

**Keywords:** photodissociation, photostability, photophysics, physical organic chemistry, photochemistry, photoactive drugs

## Abstract

We present a contemporary mechanistic description of the light-driven conversion of cyclopropenone containing enediyne (CPE) precusors to ring-opened species amenable to further Bergman cyclization and formation of stable biradical species that have been proposed for use in light-induced cancer treatment. The transformation is rationalized in terms of (purely singlet state) Norrish type-I chemistry, wherein photoinduced opening of one C–C bond in the cyclopropenone ring facilitates non-adiabatic coupling to high levels of the ground state, subsequent loss of CO and Bergman cyclization of the enediyne intermediate to the cytotoxic target biradical species. Limited investigations of substituent effects on the ensuing photochemistry serve to vindicate the experimental choices of Popik and coworkers (*J. Org. Chem*., 2005, **70**, 1297–1305). Specifically, replacing the phenyl moiety in the chosen model CPE by a 1,4-benzoquinone unit leads to a stronger, red-shifted parent absorption, and increases the exoergicity of the parent → biradical conversion.

## Introduction

Drug discovery is one of the fastest growing and most potent fields in modern day science (Elion, 1993; Takenaka, 2001; Paul et al., 2010; Rask-Andersen et al., 2011; Swinney and Anthony, 2011; Warren, 2011; Lee et al., 2012). The use and development of state-of-the-art experimental methods, coupled with molecular modeling and artificial intelligence, are revolutionizing contemporary drug discovery (Chan et al., 2019; Henstock, 2019; Mak and Pichika, 2019). These methods have also been vital for developing thorough understandings of the physiological activities of a diverse range of drugs.

Photoinitiated drug activation, wherein UV/visible light is used to drive the action of a drug, already finds use in the context of psoralens and the treatment of a range of skin conditions (Stern et al., 1980; Menter et al., 2009). The more widespread use of light-sensitive drugs, however, is still in its infancy (Farrer et al., 2010; Vernooij et al., 2018; Shi et al., 2019, 2020; Imberti et al., 2020). Such photoinitiation therapies are based on the premise that light-irradiation of a chromophore leads to formation of an excited state, with a total energy well above that of the ground state. In most cases, the total energy of the excited state species exceeds many of the activation barriers associated with ground state reactions. Electronic excitation thus provides a means of activating a reaction that is otherwise unfavorable in the ground state configuration. The excited states formed by electronic excitation can decay in many ways, both radiative (e.g., fluorescence) and non-radiative (examples of which include internal conversion, reaction (e.g., isomerization) and dissociation). Such non-radiative decay processes are usually controlled (either in part or exclusively) by conical intersections (CIs) between potential energy surfaces, which arise when two or more electronic states—e.g., an excited state and the ground state—become degenerate along a given reaction path (Domcke et al., 2004, 2011; Credo Chung et al., 2007; Xie et al., 2018). The region of degeneracy is then viewed as a CI when orthogonal nuclear motions are considered. Within the chemical physics community, CIs are now widely recognized as mediators of ultrafast internal conversion between electronic states. However, the concept, involvement and importance of CIs has yet to be fully appreciated within the wider scientific community when discussing and utilizing molecular photochemistry. This manuscript is intended, in part, to help bridge the gap between synthetic photochemistry and mechanistic photophysics—by highlighting the crucial roles of CIs in a system wherein, hitherto, such processes have been generally unrecognized and thus ignored.

Photodissociations are a class of non-radiative excited state decay processes available to molecules with labile leaving groups. The initial photoexcitation in such cases may directly populate a so-called ^1^*n*σ^*^ or ^1^πσ^*^ state, i.e., a state formed by promoting an electron from a non-bonding (*n*) or bonding π valence orbital to an antibonding σ^*^ orbital (Cronin et al., 2004; Ashfold et al., 2006, 2010; Devine et al., 2006; Nix et al., 2007; Roberts and Stavros, 2014; Marchetti et al., 2016). In many other cases, the primary photoexcitation may involve a strongly absorbing π^*^←π transition; light-driven bond fission in such cases only occurs after non-adiabatic coupling between the ππ^*^ state and an (*n*/π)σ^*^ continuum. In either scenario, the σ^*^ character lowers the overall bond order of the bond around which the orbital is localized and encourages dissociation (or predissociation) along the relevant bond-stretch coordinate.

For completeness, we note that light irradiation already finds many other roles in medicine—most notably in photodynamic therapy (PDT). PDT relies on the sensitized formation of the highly reactive excited singlet (^1^Δ_g_) state of molecular oxygen (henceforth ^1^O_2_), which can then oxidize biomolecules in the environment of cancerous cells and thereby restrict their biochemistry and eventual cell division. The sensitized formation of ^1^O_2_ requires the introduction and photoexcitation of a strongly absorbing chromophore with a high propensity for intersystem crossing (ISC). Resonant energy transfer between the photo-produced triplet state chromophore molecules and O_2_ elevates the latter to its reactive singlet state. Though effective, PDT has recognized shortcomings: e.g., (i) the eventual formation of ^1^O_2_ is indirect (relying on the excitation of, and energy transfer from, the chromophore species) and (ii) cancerous cells are usually hypoxic and successful PDT thus relies on the presence of dissolved O_2_ in the chromophore solution at the point that it is administered—thus limiting the control possible when performing PDT.

**Scheme 1:**
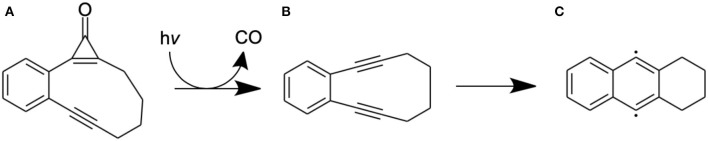
Molecular structures associated with the photoinduced dissociation of the model cyclopropenone-containing enediyne **(A)** and the subsequent Bergman rearrangement of **(B)** to the biradical **(C)**.

Light-activated chemotherapy drugs offer a direct means of targeted cancer therapy. Cyclopropenone-containing enediyne precursors are one such class of photoinitiated drugs that have been identified as potential candidates for photoinduced cancer therapy (Poloukhtine and Popik, 2005a,b; Poloukhtine et al., 2008; Pandithavidana et al., 2009). Upon photoexcitation, these molecules photodissociate, eliminating CO and forming an enediyne which then undergoes a Bergman cyclization to form a cycloalkene biradical product (Jones and Bergman, 1972; Luxon et al., 2018) that is capable of oxidizing DNA. The overall mechanism for one such precursor−2,3-benzobicyclo[8.1.0]undec-1(10)-en-4-yn-11-one—henceforth variously abbreviated as CPE and as **A**—is illustrated in Scheme 1.

The present manuscript reports the use of contemporary multi-reference electronic structure methods to explore mechanistic details of the reaction paths that lead to the observed (Poloukhtine and Popik, 2005a) photoinduced CO elimination from CPE and the ensuing Bergman rearrangement of **B** to **C** and how these processes might be affected by selected changes to the phenyl ring.

## Computational Methodology

All calculations were conducted for the isolated (i.e., gas phase) molecule. The ground state minimum energy geometry of CPE (**A**) was optimized using the Coulomb-Attenuated Model Becke-3rd parameter-Lee-Yang-Parr (CAM-B3LYP) functional (Yanai et al., 2004) of Density Functional Theory (DFT), coupled to the 6-31G(d) Pople basis set (Hehre et al., 1970). Since the reaction of interest (Scheme 1) starts with photoinduced elimination of CO, two-dimensional potential energy (PE) profiles of the ground and first excited electronic states of this CPE along the two C–CO stretch coordinates of the cyclopropenone moiety were first investigated using Time-Dependent Density Functional Theory (TD-DFT) (Van Caillie and Amos, 2000; Furche and Ahlrichs, 2002; Scalmani et al., 2006), with the above functional and basis set. As shown in Supplementary Figure 1 in the Electronic Supplementary Information (ESI), these test calculations returned a maximum on the excited state PE surface when both C–CO bond distances were extended in tandem, but minima when either C–CO bond was extended while the other was held fixed. This hinted that the photoinduced CO elimination involves an initial ring-opening prior to release of the CO moiety.

Guided by these computations, the lowest triplet spin configuration of the ring-opened species formed by breaking the C–C bond nearer the phenyl ring was optimized at the CAM-B3LYP/6-31G(d) level of theory and used as a proxy for the singlet ring-opened structure and energy (since calculations for the ground singlet state inevitably resulted in reformation of the ring-closed CPE molecule).

PE profiles connecting the ground state CPE structure to the optimized ring-opened biradical structure and then onwards to the bicyclic structure **B** were then computed using the complete active space second-order perturbation theory (CASPT2) method, (Roos et al., 1982; Andersson et al., 1990, 1992; Park and Shiozaki, 2017) based on a state-averaged complete active space self-consistent field (SA-CASSCF) reference wavefunction (comprising five singlet and four triplet states) and coupled to the cc-pVDZ basis set (Dunning, 1989). The PE path was constructed using successive linear interpolations in internal coordinates (LIICs) and each LIIC was checked to ensure that the intermediate geometries represented a sensible interpolation between the optimized ground state CPE, the ring-opened species and then **B**. All of these CASSCF and CASPT2 computations employed an active space involving 10 electrons distributed in 10 orbitals—the five highest occupied (**1**–**5**) and the five lowest virtual (**6**–**10**) orbitals shown in Supplementary Figure 2 of the ESI, where **5** and **6** are, respectively, the highest occupied molecular orbital (HOMO) and lowest unoccupied molecular orbital (LUMO) in the ground state. A further more limited set of PE profiles mapping the Bergman rearrangement of **B** to the biradical **C** were then computed using CASPT2/cc-pVDZ methods, based on a SA-CASSCF reference wavefunction comprising just two singlet and two triplet states (since our primary interest was in determining the magnitude of any energy barrier to **B** → **C** conversion on the ground state PE surface) and an active space comprising 10 electrons in 10 orbitals (shown in Supplementary Figure 3 of the ESI where, again, orbitals **5** and **6** are, respectively the HOMO and LUMO of the ground state molecule). In all cases, an imaginary level shift of 0.3 *E*_h_ was used to aid convergence and mitigate against the involvement of intruder states.

Vertical excitation energies (VEEs) and transition dipole moments were extracted from the associated CASSCF/CASPT2 calculations at the ground state equilibrium geometries of **A** (and **B**) to derive oscillator strengths (*f*) for transitions to the first few excited states (just the respective S_1_ states in the case of **B**) using Equation (1).

(1) $$\begin{array}{l} f=\frac{2}{3}\left(E_{i}-E_{0}\right)\cdot \sum_{\alpha =x,y,z}|\mu_{0i}{|}_{\alpha }^{2}\;, \end{array}$$

where *E*_*i*_ and *E*_0_ are, respectively, the energies of the excited state of interest and the ground state and the μ_0*i*_ are the associated transition dipole moments along α (α = *x, y*, and *z*). The different *x* and *y* coordinates used in describing the transitions in **A** and **B** are shown by the red and green arrows in Supplementary Figures 2, 3; the *z* coordinate in both cases is out of the page.

Additional CASPT2 computations using the same basis set and imaginary level shift together with a (10,8) active space were undertaken for three analogs of CPE wherein the phenyl ring had been substituted symmetrically with methoxy (OMe) or cyano (CN) groups in the 1- and 4-positions, or replaced with a 1,4-benzoquinone ring, in order to explore substituent effects on the VEEs and transition strengths. Supplementary Figure 4 in the ESI shows the highest occupied and lowest virtual orbitals in the ground state of each system. These variants will henceforth be referred to as 1,4-MeO-CPE, 1,4-CN-CPE and 1,4-O=CPE. The latter was selected as (2,3-dimethyl substituted) 1,4-O=CPE has been synthesized (from the dimethylated analog of 1,4-MeO-CPE), shown to form the corresponding enediyne by photoinduced CO elimination and touted as a potential cytotoxin (Poloukhtine and Popik, 2005b). The other two substituted CPEs were selected to compare and contrast the effect of strongly π-accepting (i.e., CN) and π-donating (i.e., MeO) substitutents. The ground state energies of the **A**, **B**, and **C** analogs of 1,4-MeO-CPE, 1,4-CN-CPE and 1,4-O=CPE, relative to bare CPE, were optimized using the high-level CBS-QB3 method (Saracino et al., 2003; Cysewski, 2007; Rayne and Forest, 2010). All DFT and CBS-QB3 calculations were performed using Gaussian 16 (Frisch et al., 2016), while all CASSCF/CASPT2 computations were undertaken in Molpro 2018 (Werner et al., 2018).

## Results and Discussion

### Ground State Structure and Vertical Excitation Energies

**Figure 1:**
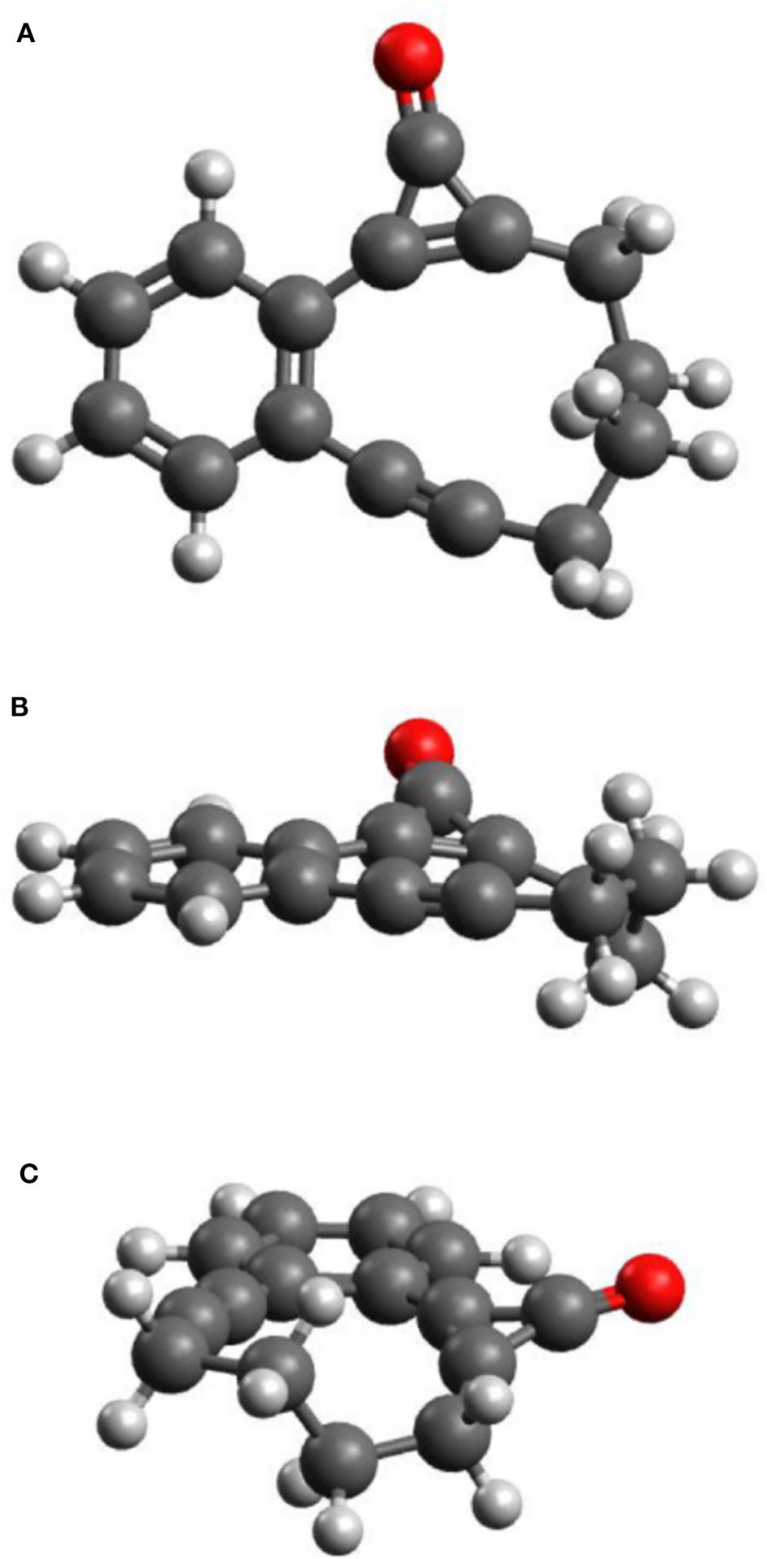
Plan **(A)** and side views **(B,C)** of the optimized molecular structure of CPE in its ground electronic state.

Figures 1A–C display plan and side-on views of the ground state optimized geometry of CPE. The phenyl, cyclopropenone and alkyne moieties lie in a common plane, while the C_4_H_8_ alkyl link adopts a non-planar configuration. Given the cyclic nature of CPE, the geometry around the cyclopropenone unit can be viewed as a strained form of bare cyclopropenone—with an intra-large-ring C–C=C bond angle of ~175° (*cf*. an H–C=C bond angle of ~125° in cyclopropenone)–and the alkyne moiety adopts a strained form of butyne.

Table 1 lists the vertical excitation energies (VEEs) to the first few excited singlet and triplet states of CPE and the dominant orbital promotions involved in the first two singlet excitations. The calculated VEE of the more intense S_2_-S_0_ transition agrees well with that reported for CPE in Poloukhtine and Popik (2005a) and Poloukhtine and Popik (2006a), but the calculated S_1_-S_0_ transition energy is below that associated with the observed weaker, longer wavelength absorption maximum.

**Table 1:** Vertical excitation energies (VEEs) and oscillator strengths (*f*) of transitions to the first few singlet and triplet excited states of CPE, calculated at the CASPT2/AVDZ level of theory.

**Transition**	**Dominant orbital promotions**	**VEE/eV**	***f***
S_1_-S_0_	6←5 (0.86); 7←5 (0.16); 8←5 (0.03)	3.00	0.0053
S_2_-S_0_	6←3 (0.55); 7←4 (0.33); 6←4 (0.30)	4.43	0.0127
T_1_-S_0_	6←4 (0.70); 6←5 (0.36); 7←4 (0.30)	2.87	0
T_2_-S_0_	6←5 (0.79); 6←4 (0.32); 7←5 (0.19)	3.09	0
T_3_-S_0_	6←3 (0.67); 7←4 (0.51); 6←2 (0.22)	4.03	0

*The second column details the orbital (see Supplementary Figure 2) promotions that make the larger contributions (expressed as coefficients in parentheses) to the respective excitations*.

Reference to Table 1 and the orbitals displayed in Supplementary Figure 2 shows that the first excited singlet (S_1_) state of CPE has ππ^*^ character and is formed by electron promotion between orbitals that include π-electron density distributed over the phenyl ring, cyclopropenone and alkyne moieties. The S_2_ state has both *n*π^*^ and ππ^*^ character and is formed by promotion from a ‘mixed’ bonding orbital that involves a non-bonding *p*σ component (localized on the O atom) and a π-bonding component that is mainly localized on the phenyl ring but also conjugates across the C=C bond of the cyclopropenone unit. The participating *n/*π and π^*^ orbitals show significant spatial overlap, reflected in a calculated S_2_-S_0_ oscillator strength some 2.5× larger than that of the S_1_-S_0_ transition (Table 1). This suggests a significant contribution from the phenyl-ring centered π-bonding region and, for brevity, we will henceforth refer to S_2_ as a ππ^*^ excited state also.

For completeness, Table 1 also lists the calculated VEEs to the first three triplet states. Excitations to these states from the singlet ground state are spin-forbidden and thus calculated to have zero oscillator strength. We also note the near degeneracy of the T_1_ and T_2_ states in the vertical region, and that the T_2_ and S_1_ states share a common dominant electronic configuration at this geometry.

### Reaction Path Following Photoexcitation

**Figure 2:**
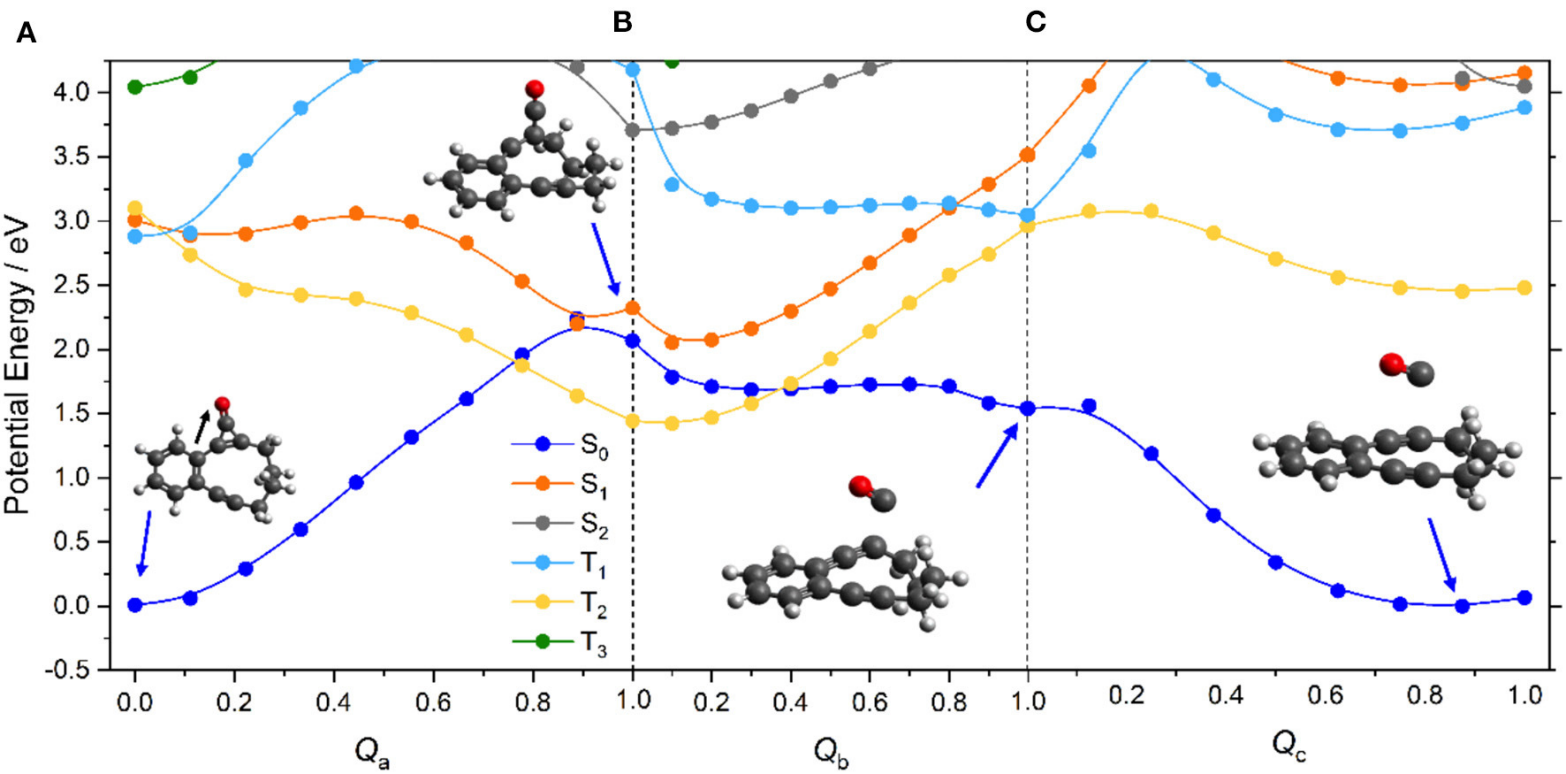
CASPT2 potential energy profiles for the ground and first few excited states of CPE along the sequence of dimensionless LIICs (panels (a) through (c) as discussed in the text) involved in the photoinduced conversion of **A** → **B** (+ CO) products, with the molecular geometries at various critical points depicted. The color coding of the states shown in the inset in panel **(A)** defines the state ordering at *Q*_a_ = 0. Note, the energetic ordering of the states labeled T_1_ and T_2_ switches once *Q*_a_ > 0, and thereafter the adiabatic PE profiles of the T_1_ and T_2_ states are described by, respectively, the yellow and pale blue curves—here and in later figures.

Figure 2 displays the PE profiles along the reaction path depicted in Scheme 1. Panel (a) shows the profiles returned from a LIIC along the dimensionless coordinate *Q*_a_ connecting the minimum energy geometries of ground state CPE and the (triplet) intermediate formed via ring-opening of the cyclopropenone unit. As noted previously (Poloukhtine and Popik, 2006b), the alternative ring-opened intermediate that would be formed by cleaving the other C–C bond in the cyclopropenone unit (i.e., the bond further from the phenyl ring) lies at significantly higher energy (~0.35 eV in the present calculations) and is not considered further. As such, *Q*_a_ is predominantly the C–C bond stretch indicated by the black arrow in Figure 2A. Along this path, the PE of the S_0_ state shows a steady increase—as would be expected for the rupture of a C–C σ-bond. The PE of the S_1_ state, in contrast, shows a small barrier and then decreases along this ring-opening coordinate. At this point, it is important to recall that LIIC pathways must, by construction, return upper estimates of any barriers that would be derived by locating and optimizing the true transition states, and we henceforth assume minimal barrier to distortion along *Q*_a_ on the S_1_ PE surface. The topography of the S_1_ potential can be understood by recognizing that the electronic configuration of the S_1_ state (predominantly ππ^*^ in the vertical region) gains increasing *n*σ^*^ character upon C–C bond extension. The decline in the S_1_ state PE, together with the progressive increase in the S_0_ state PE, leads to an inevitable crossing at *Q*_a_ = 0.9. This region of conical intersection is likely to encourage non-adiabatic coupling between the S_1_ and S_0_ states, i.e., to facilitate internal conversion to high vibrational levels of the S_0_ state of CPE. These topological details serve to reinforce the earlier conclusion that the light-induced decarbonylation in cyclopropenones is a stepwise process initiated on the S_1_ PE surface (Poloukhtine and Popik, 2006b).

This photoinduced ring opening can be viewed as another example of a singlet-mediated Norrish type I α-C–C bond cleavage in a ketone. Norrish type I reactions have traditionally been assumed to start with efficient S_1_ → T_1_ ISC (Norrish and Bamford, 1936, 1937; Diau et al., 2001, 2002), and indeed, reference to Figure 2A suggests that S_1_ → T_1_ ISC in CPE could constitute another route to the ring-opened species. However, the recent literature includes a growing number of predictions and/or demonstrations of purely singlet-state enabled fission of the C–C bond in the α-position (relative to the carbonyl group) (Maeda et al., 2010; Nádasdi et al., 2010; Marchetti et al., 2019), notably in strained cyclic ketones like cyclobutanone (Xia et al., 2015; Shemesh et al., 2016; Kao et al., 2020). In all such cases investigated to date, the C–C=O moiety remaining after photochemical cleavage of the other α-C–C bond is predicted to be near linear at the CI (Marchetti et al., 2019)—as is found in the present case also (see Figure 2). The photoinduced C–C bond fission in CPE is another such example, not least because—as we now show—Scheme 1 requires further chemistry that can only occur on the S_0_ PE surface. As Supplementary Figure 5 in the ESI shows, the Mulliken charge distributions for the optimized ring-opened structure of the isolated molecule in its T_1_ state and for the S_0_ state molecule at that same geometry are similar, and far from limiting biradical or zwitterionic in character. Clearly, however, the degree of charge separation is likely to be different in solution and to be solvent-dependent.

The highly vibrationally-excited S_0_ molecules formed by non-adiabatic coupling from the S_1_ state may relax to reform the starting CPE molecule or follow a rival path on the S_0_ PE surface. Given the experimental observations (Poloukhtine and Popik, 2005a, 2006b), one possible rival path must involve CO loss from the ring-opened biradical to form intermediate **B** in Scheme 1. To model this process, PE profiles were first computed simply by stepping the C–CO bond stretch coordinate (*R*_C−CO_), but this revealed an obvious discontinuity in the S_0_ and T_1_ PE profiles at *R*_C−CO_ ~ 1.8 Å. This was traced to a localized linear to bent change in the geometry of the C–C=O unit, that we assume to be a signature of the onset of a π-stacking interaction between the emerging CO product and the alkyne group. Thus, the overall C–CO bond fission following opening of the cyclopropenone ring was modeled using two successive LIICs—*Q*_b_, to cover the region from the minimum energy geometry of the triplet ring-opened structure to the relaxed bent geometry at *R*_C−CO_ ~1.8 Å, then *Q*_c_, from the bent geometry at *R*_C−CO_ ~1.8 Å to long range.

The S_0_ and excited state PE profiles associated with *Q*_b_ in Figure 2B show several features of note. The PE profile of the T_1_ state (which is the most stable configuration of the ring-opened species) increases steadily. Unsurprisingly, given that the S_1_ and T_1_ states at these geometries are well-approximated as spin-flip variations of the same electronic configuration, the S_1_ PE profile shows a similar increase. The PE of the S_0_ state, in contrast, declines steadily along *Q*_b_. This finding can be understood by inspecting the HOMO of the ring-opened species, which shows antibonding π^*^ character around the C–CO bond. Occupancy of this orbital reduces the C–CO bond strength and, as with the initial ring-opening step, this HOMO gains progressive σ^*^ character upon C–CO bond extension—leading to eventual bond fission. Figure 2C shows the corresponding PE profiles along *Q*_c_. Of particular relevance to the current narrative, the PE profile of the S_0_ state shows a continued steady decrease *en route* to forming the alkyne intermediate (structure **B** in Scheme 1).

**Figure 3:**
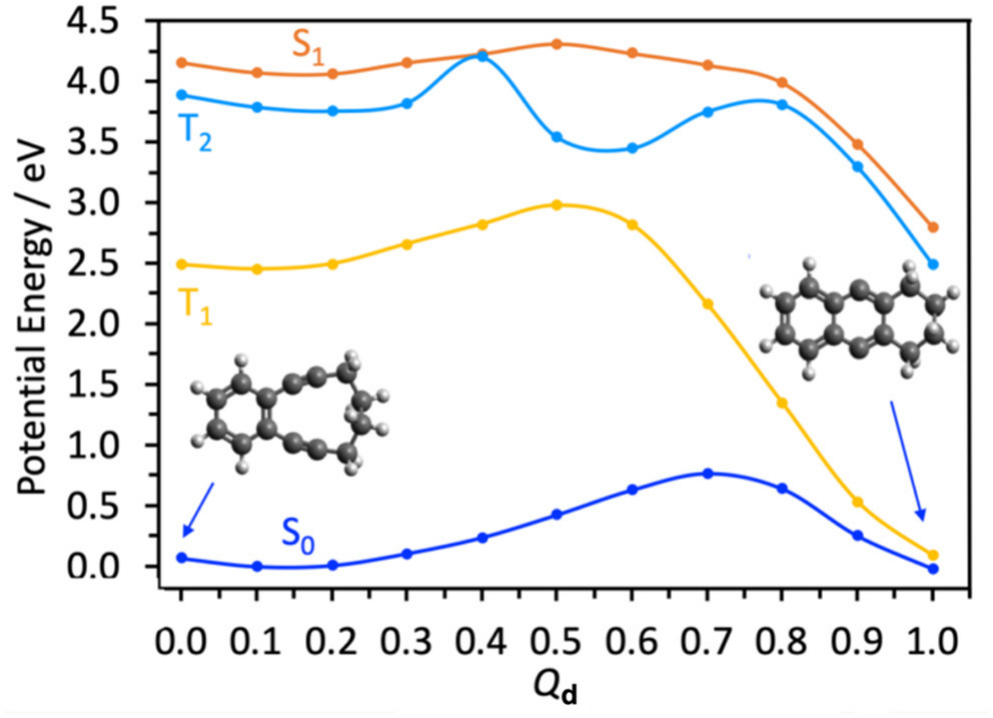
CASPT2 potential energy profiles for the ground and first three excited states along the dimensionless LIIC *Q*_d_ describing the **B** → **C** conversion.

Scheme 1 shows this alkyne intermediate undergoing a Bergman cyclization to form a ring-closed bicyclic biradical (structure **C**). Figure 3 shows the calculated (by CASPT2) energy profile for this final step on the S_0_ PE surface, along a LIIC (*Q*_d_) linking structures **B** and **C**. This shows the di-alkyne intermediate **B** and the tricyclic biradical species **C** having essentially the same minimum energies, and separated by an intervening energy barrier with Δ*E* ~0.8 eV. Reference to Figure 2 shows that the minimum energy of **A** is also very similar to that of the **B** (+CO) limit. Noting the earlier comment that any energy barrier returned by a LIIC calculation will necessarily be an upper estimate of the true transition state energy and that the calculated barrier energy in Figure 3 is well below that of the CI between the S_1_ and S_0_ PE surfaces of CPE (Figure 2A) suggests that both **B** and **C** could be thermodynamically viable products following photoexcitation of CPE, within which population may partition—consistent with the experimental observation of **B** formation [and the ensuing Bergman cyclisation to **C** at quite modest temperatures (84°C)] (Poloukhtine and Popik, 2005a).

Traditional organic photosyntheses such as that used to drive the transformation **A** → **C** employ prolonged illumination, so it is also prudent to consider possible photoinduced chemistry of the product **B** (Poloukhtine and Popik, 2005a,b, 2006a,b; Poloukhtine et al., 2008). Table 2 lists the calculated VEEs to the S_1_, T_1_, and T_2_ states of **B** (all of which are best viewed as having ππ^*^ character) and the S_1_-S_0_ oscillator strength and dominant contributing promotions based on the orbitals displayed in Supplementary Figure 3. Figure 3 shows the calculated PE profiles of these three excited states along *Q*_d_. As Table 2 shows, the S_1_-S_0_ transition of **B** is predicted to have a similar oscillator strength to that of CPE but to have an appreciably larger VEE—which matches reasonably with the absorption maximum of the product formed by 300 nm irradiation of CPE reported in Poloukhtine and Popik (2005a).

**Table 2:** Vertical excitation energies (VEEs) and oscillator strengths (*f*) of transitions to the lowest singlet and triplet excited states of **B**, calculated at the CASPT2/AVDZ level of theory.

**Transition**	**Dominant orbital promotions**	**VEE/eV**	***f***
S_1_-S_0_	6←4 (0.61); 7←5 (0.52); 6←5 (0.18)	4.09	0.0050
T_1_-S_0_	6←5 (0.89); 7←4 (0.18); 6←5+[7←4]^2^ (0.13)	2.42	0
T_2_-S_0_	6←4 (0.61); 7←5 (0.52); 6←5 (0.12)	3.82	0

*The second column details the three orbital (see Supplementary Figure 3) promotions that make the largest contribution (expressed as coefficients in parentheses) to the respective excitations. [ ]^2^ implies a double excitation*.

The PE profiles shown in Figure 3 hint that secondary photoexcitation of **B** could feasibly aid **C** formation. Specifically, we note the near degeneracy of the S_1_ and T_2_ states at small *Q*_d_ and the possibility of strong non-adiabatic coupling around *Q*_d_ ~ 0.4, where the S_1_ potential samples a region of configuration space that supports a conical intersection between the T_2_ and (not shown) T_3_ potentials. Any S_1_ population undergoing (spin-orbit induced) transfer to the T_2_ potential in this region could thereafter follow an energetically “down-hill” path via the T_2_/T_1_ conical intersection at *Q*_d_ ~ 0.55 and relax into the potential well of the low-lying triplet form of **C**. Higher level theory could inform on the possible importance of spin-orbit enabled transfer to the T_1_ potential, but electron paramagnetic resonance (EPR) studies as a function of illumination time might be the best way of exploring the relative importances of one vs. sequential two photon production of **C** by near UV irradiation under synthetically relevant solution phase conditions.

### Substituent Effects on the Electronic Absorption and Potential Reactivity of CPEs

Table 3 compares the VEEs, the *x, y*, and *z* components of the respective transition dipole moments and the overall oscillator strengths for forming the S_1_ and S_2_ states of bare CPE and the 1,4-MeO-CPE, 1,4-O=CPE and 1,4-CN-CPE analogs returned by the CASPT2 calculations. The orbital promotions that make the greatest contributions to the S_1_-S_0_ and S_2_-S_0_ transitions in each of the substituted molecules are included in Supplementary Figure 4 in the ESI.

**Table 3:** Vertical excitation energies (VEEs), *x, y*, and *z* components of the respective transition dipole moments [TDMs (in atomic units)] and oscillator strengths (*f*) for the S_1_-S_0_ and S_2_-S_0_ transitions of (a) bare CPE, (b) 1,4-methoxy substituted CPE, (c) the 1,4-benzoquinone derivative, and (d) 1,4-cyano substituted CPE, calculated at the CASPT2/AVDZ level of theory.

**Molecule**	**Transition**	**VEE/eV**	**TDM*_***x***_***	**TDM*_***y***_***	**TDM*_***z***_***	***f***
(a) 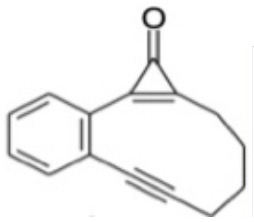	S_1_-S_0_	3.00	−0.070	0.220	0.136	0.0053
	S_2_-S_0_	4.43	0.319	0.120	0.024	0.0127
(b) 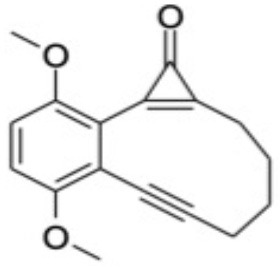	S_1_-S_0_	3.25	0.872	−0.047	0.067	0.0706
	S_2_-S_0_	3.75	0.260	−0.581	0.433	0.0325
(c) 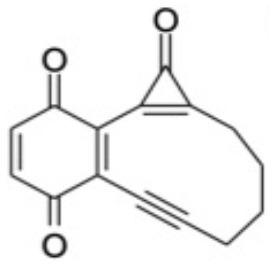	S_1_-S_0_	2.54	0.308	−0.781	0.086	0.0443
	S_2_-S_0_	3.81	−0.708	1.610	0.039	0.2887
(d) 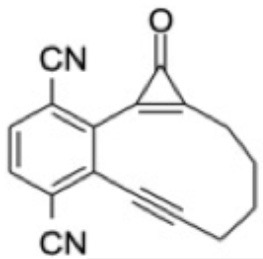	S_1_-S_0_	3.25	0.256	−0.253	0.124	0.0115
	S_2_-S_0_	3.68	−0.371	−0.252	−0.021	0.0182

Even a cursory inspection of Table 3 suffices to show that such symmetric changes at the 1- and 4-positions on the phenyl ring have a substantial effect on the electronic absorption of CPE. All are predicted to enhance the S_1_-S_0_ oscillator strength (*cf*. bare CPE), most strikingly in the cases of 1,4-MeO-CPE and the 1,4-benzoquinone derivative. Each involves net loss of electron density from the cyclopropenone carbonyl group (reflected in the negative TDM_*y*_ values in Table 3); the S_1_ molecules have some zwitterionic character—in accord with conclusions reached in earlier theoretical studies of alkyl- or aryl-substituted cyclopropenones (Poloukhtine and Popik, 2006b). Substituting the phenyl ring with π-accepting (i.e., CN) or π-donating (i.e., MeO) groups increases the VEE of the S_1_-S_0_ transition (*cf*. bare CPE), but the enhanced conjugation introduced by replacing the phenyl ring by a 1,4-benzoquinone ring causes an obvious red-shift in the S_1_-S_0_ absorption.

The effects on the S_2_-S_0_ transitions are more substituent specific, with the calculated TDMs suggesting accumulation of electron density on the substituted phenyl ring in the case of 1,4-MeO-CPE and on the cyclopropenone sub-unit in the case of 1,4-O=CPE. In all cases, substitution is predicted to reduce the VEE of the S_2_-S_0_ transition (*cf*. bare CPE)—in accord with the available experimental data for 1,4-O=CPE and CPE (Poloukhtine and Popik, 2005a,b)

Table 4 shows the effect of substituents on the relative stabilities of the bicyclic enediyne and the Bergman cyclized product (henceforth labeled **B**′ and **C**′) relative to the corresponding **B** and **C** products from photoexcitation of bare CPE. CO elimination (i.e., **A**′ → **B**′) is favored, energetically, by either CN substitution or by replacing the phenyl ring with a 1,4-benzoquinone unit, whereas 1,4-MeO substitution leads to a **B**′ product that is marginally less stable than for bare CPE. The former stabilizations can be plausibly understood by considering the extended π-systems in the enediyne product. Thermodynamically, 1,4-O=CPE is in a class of its own with regard to the final **B**′ → **C**′ electrocyclization reaction, consistent with its adoption as a precursor of choice by (Poloukhtine and Popik, 2006a).

**Table 4:** CBS-QB3 calculated enthalpy changes associated with the respective **A**′ → **B**′ and **B**′ → **C**′ conversions in (b) 1,4-methoxy substituted CPE, (c) the 1,4-benzoquinone derivative, and (d) 1,4-cyano substituted CPE, each referenced to the corresponding **A** → **B** and **B** → **C** conversions in bare CPE (a).

**Molecule**	**Δ*****H*** **/ eV**	
	**Δ*H*_(A′ → B′)_ − Δ*H*_(A → B)_**	**Δ*H*_(B′ → C′)_ − Δ*H*_(B → C)_**
(a) 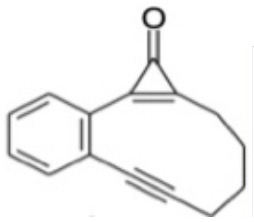	0	0
(b) 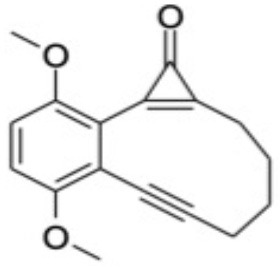	0.025	−0.014
(c) 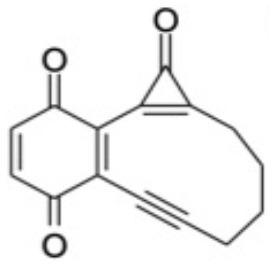	−0.080	−0.407
(d) 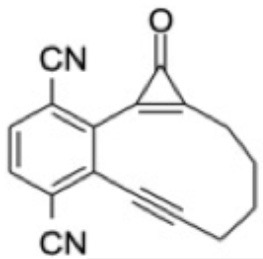	−0.142	−0.001

## Conclusions

This study offers a contemporary mechanistic description of the light-driven conversion of cyclopropenone containing enediyne precusors like CPE to ring-opened species amenable to further Bergman cyclization, resulting in stable biradical species that have been proposed for future use in light-induced cancer treatment (Poloukhtine and Popik, 2005a,b, Poloukhtine and Popik, 2006a; Poloukhtine et al., 2008). The initial photoinduced cleavage of the C–C bond in the cyclopropenone ring closer to the phenyl group is shown to follow Norrish type-I chemistry. In the present case, this can be accommodated by purely singlet-state chemistry, i.e., efficient non-adiabatic coupling from the photo-prepared S_1_ state to high levels of the ground (S_0_) PE surface, upon which subsequent nuclear rearrangements lead to CO loss and formation of **B**, followed by the electrocyclization of **B** to yield the cytotoxic target biradical species **C**.

Viewed from the perspective of the photoexcited A^*^ precursor in Scheme 1, the reactions through to ground state **B** (and **C**) products are exoergic processes, the efficiencies of which are likely to be sensitive to the relative rates of reaction and internal energy loss to the solvent. Deliberate (or otherwise) secondary photoexcitation of the enediyne intermediate **B** is suggested as a way of boosting the yield of the target diradical species **C**. The present limited investigation of the effects of chemically modifying the phenyl moiety in **A** serves to vindicate the experimental choices made by Popik and coworkers (Poloukhtine and Popik, 2006a). Specifically, replacing the phenyl moiety in CPE by a 1,4-benzoquinone unit is shown to both red-shift and boost the absorption of the **A**′ precursor and to increase the exoergicity of the required **A**′ → **C**′ conversion—complementing current efforts to use visible-light-responsive photocatalysts to trigger alkyne generation from (non-visible light absorbing) cyclopropenones (Mishiro et al., 2019).

## Data Availability Statement

The raw data supporting the conclusions of this article will be made available by the authors, without undue reservation.

## Author Contributions

TK and MA designed the project. SL, BM and TK undertook the underlying data. All authors contributed to drafting the manuscript.

## Conflict of Interest

The authors declare that the research was conducted in the absence of any commercial or financial relationships that could be construed as a potential conflict of interest. The handling editor declared a past collaboration with the authors TK and MA.
