# Supplementary material for: The Role of Norrish Type-I Chemistry in Photoactive Drugs: An ab initio Study of a Cyclopropenone-Enediyne Drug Precursor
Source: Front Chem. 2020 Dec 22;8:596590. doi: 10.3389/fchem.2020.596590 (PMC7793749; doi:10.3389/fchem.2020.596590)
Supplement: Supplementary file 1 [file Data_Sheet_1.PDF]

Electronic Supplementary Information for:

The Role of Norrish Type-I Chemistry in Photoactive Drugs: An *ab initio* Study of a Cyclopropenone-Enediyne Drug Precursor

Spencer J. Léger[†,‡], Barbara Marchetti[†], Michael N. R. Ashfold[#] and Tolga N. V. Karsili[†,*]

[†]*Department of Chemistry, University of Louisiana at Lafayette, Louisiana, 70503, USA*

[‡]*Department of Chemical Engineering, University of Louisiana at Lafayette, Louisiana, 70503, USA*

[#]*School of Chemistry, University of Bristol, Bristol, BS8 1TS, UK*

Figure S1: TD-DFT calculated S_0 (black) and S_1 (red) PE profiles starting from the ground state minimum geometry (point 1) along LIICs describing fission of the single C–CO bond (a) nearer to and (b) further from the phenyl ring and (c) for the simultaneous extension of both C–CO bonds in **A**. The geometries at the points labelled 10 in (a), (b) and (c) were defined as follows: (a) the optimized ring opened structure in the T_1 state (i.e. $Q_a = 1$ in Fig. 2(a)); (b) the optimized ring opened structure (again in the T_1 state) achieved by extending the other C–C bond in the cyclopropenone ring – again optimized at the CAM-B3LYP/6-31G(d) level of theory; and (c) the structure corresponding to $Q_c = 1$ in Fig. 2(c).

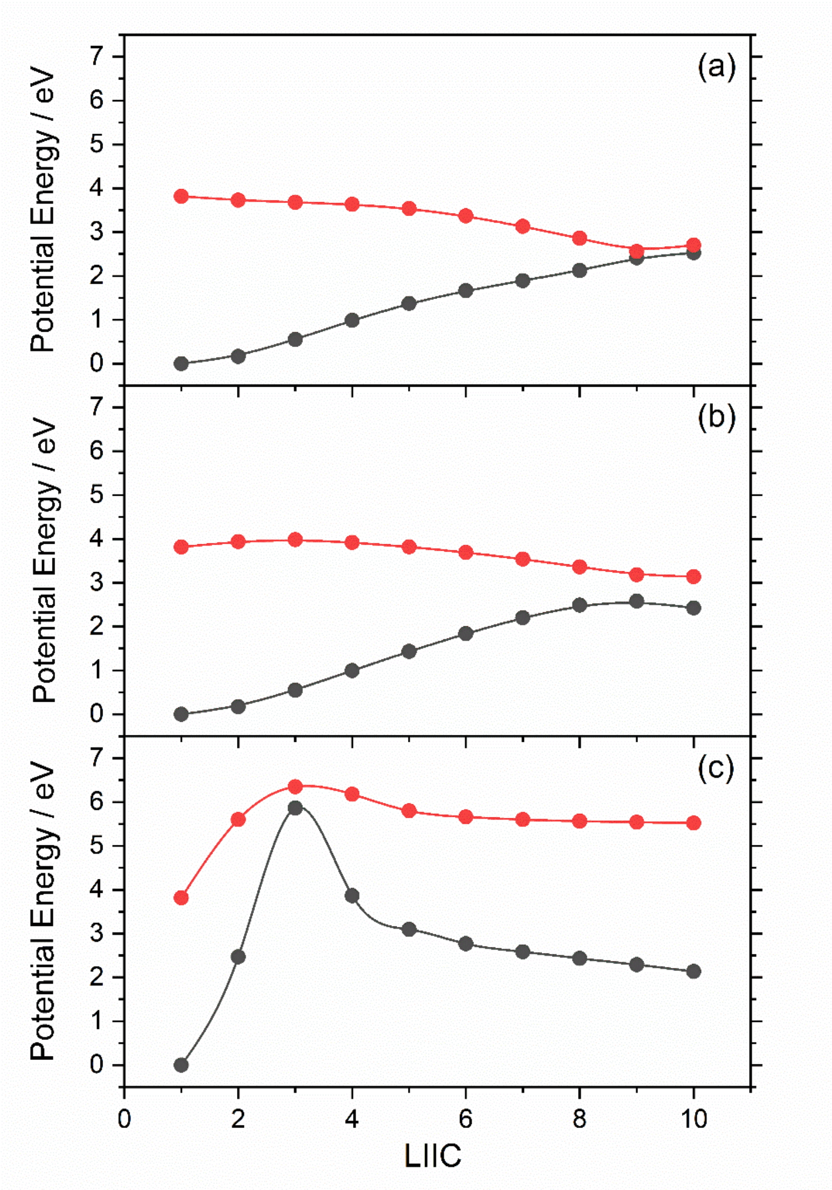

Figure S2: Active orbitals used in the CASSCF computations of structure **A**, with orbitals 5 and 6 defining, respectively, the HOMO and LUMO in the ground state configuration and the superposed red and green arrows defining the +*x* and +*y* directions, respectively, from the centre of mass.

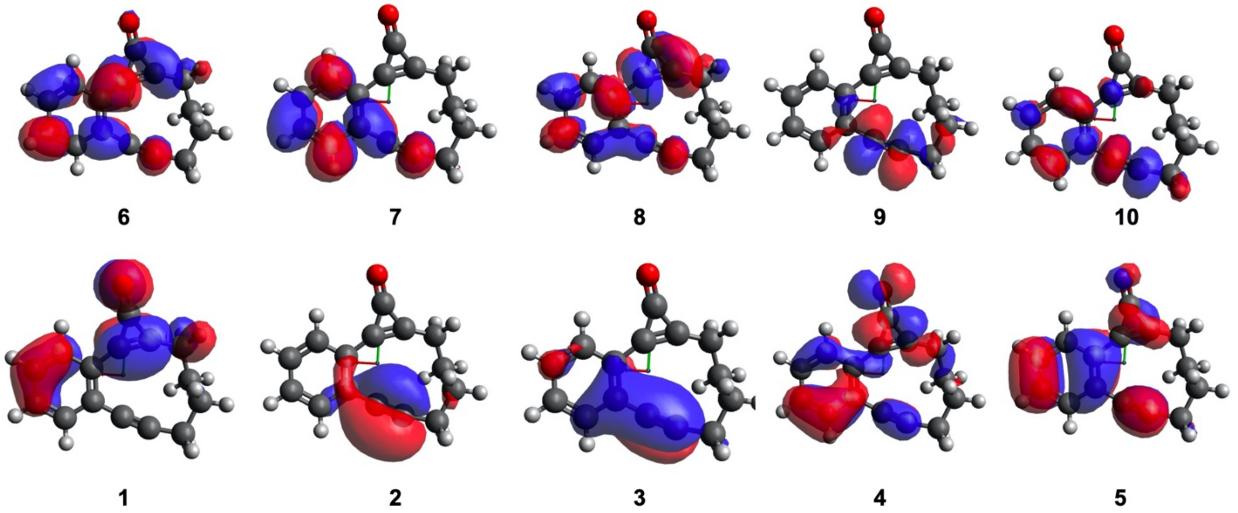

Figure S3: Active orbitals used in the CASSCF computations of structure **B**, with orbitals 5 and 6 defining, respectively, the HOMO and LUMO in the ground state configuration and the superposed red and green arrows defining the $+x$ and $+y$ directions, respectively, from the centre of mass.

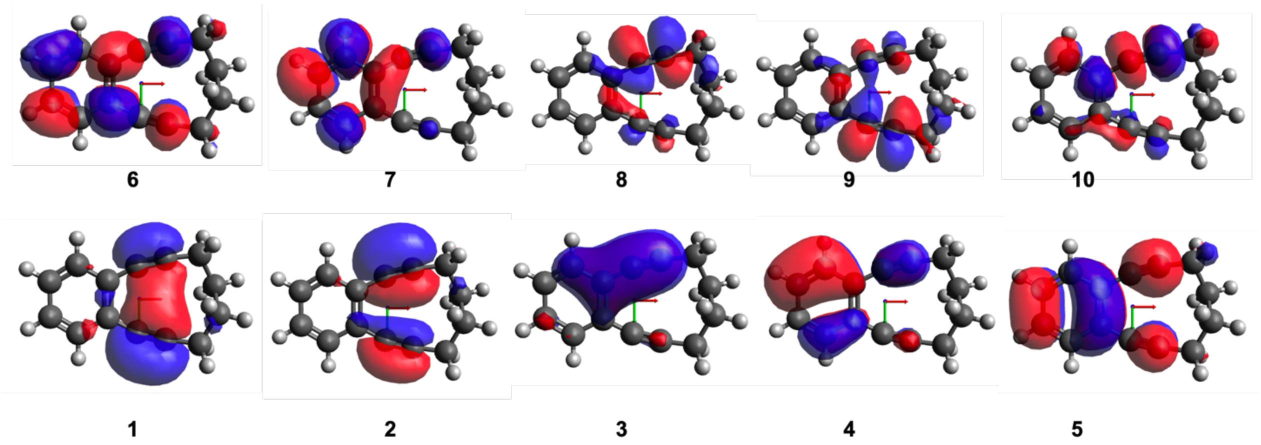

Figure S4: Dominant orbital promotions for 1,4-MeO-CPE, 1,4-O=CPE and 1,4-CN-CPE. The blue and red arrows show the dominant contributors to, respectively, the S_1-S_0 and S_2-S_0 transitions along with their associated coefficients. The dashed black line represents the separation between the occupied (below the line) and unoccupied (above the line) orbitals in the ground state and the red and green arrows define the +*x* and +*y* directions used in defining the TDM components in Table 3.

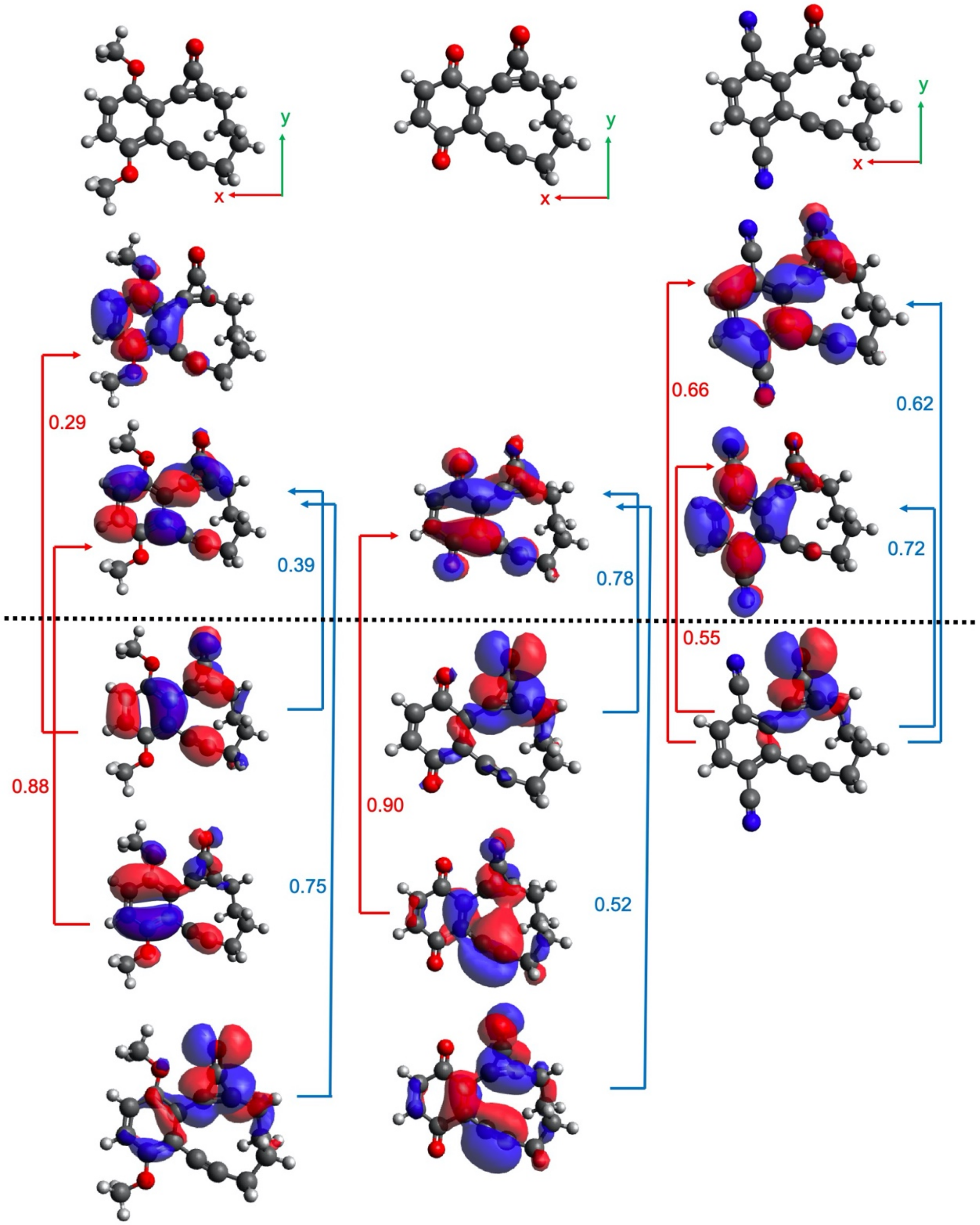

Figure S5: Mulliken charge distribution at (a) the optimized ring-opened structure in the T_1 state and (b) this same ring-opened structure, the equilibrium ring-closed structure and one intermediate geometry on the S_0 PE surface.

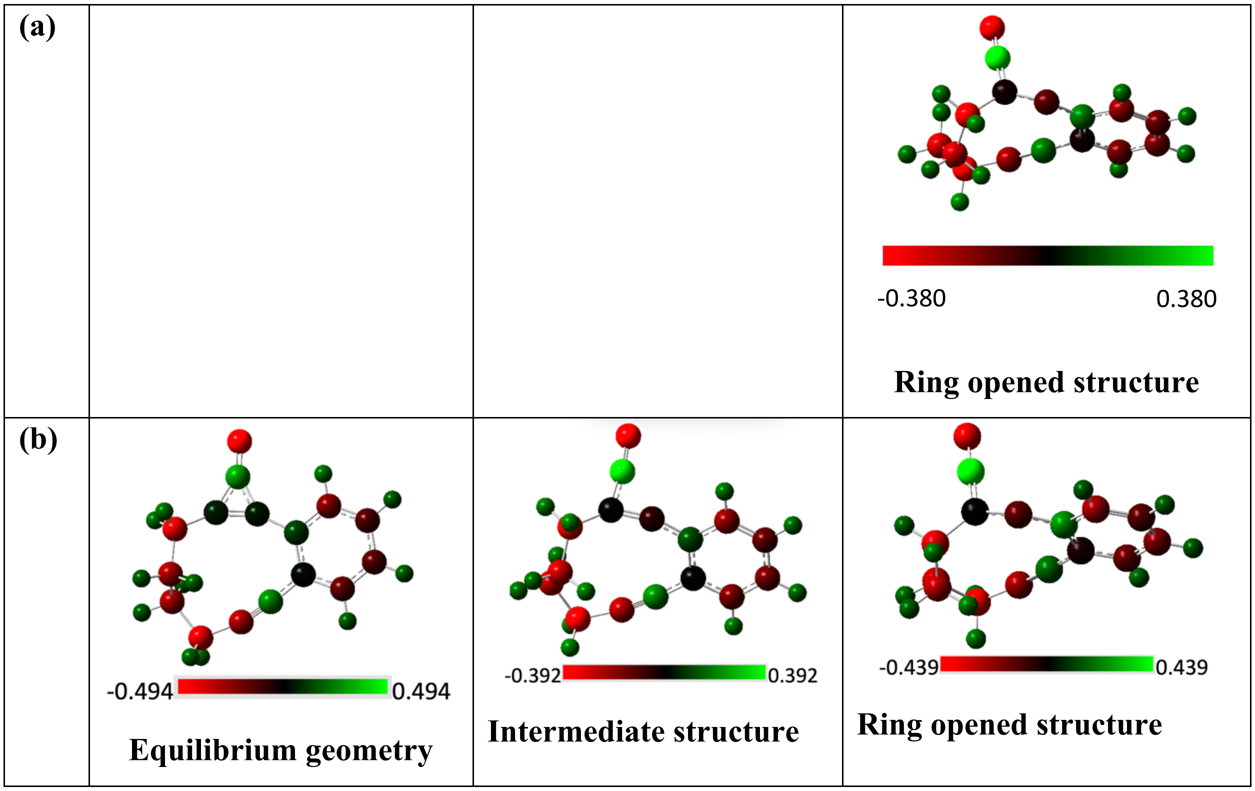

Cartesian coordinates (in Å) for:

CPE Structure A

C	3.63408208	-1.38491297	0.15816900
C	2.37329507	-1.96143305	0.06614300
C	1.22165799	-1.16393495	-0.00879300
C	1.35780597	0.24863100	0.01163900
C	2.64414096	0.80836499	0.07729800
C	3.77241111	0.00317200	0.16117100
H	4.51099920	-2.01940107	0.21745899
H	4.75565100	0.45465201	0.22037700
C	0.25234100	1.18730795	-0.05726900
C	-1.04891896	1.57678199	0.10409300
C	-2.45700002	1.37327003	0.55279100
H	-3.10448408	1.87059903	-0.18002801
H	-2.55160904	1.98444605	1.46054995
C	-2.96905088	-0.04619400	0.84008902
H	-2.23139310	-0.56655502	1.45652401
H	-3.86988091	0.04587300	1.45377004
C	-3.29248095	-0.88474602	-0.41972601
H	-4.37456608	-0.99392700	-0.53789300
H	-2.93022799	-0.37065300	-1.31489396
C	-2.64569592	-2.28938007	-0.39246899
H	-2.90261006	-2.84266305	-1.30187094
H	-3.03443599	-2.87465501	0.44948599
C	-1.20278203	-2.12935305	-0.27309999
C	-0.06816900	-1.74265099	-0.13868301
C	-0.08856600	2.55708194	-0.28037500
O	0.22928600	3.69387698	-0.55469698
H	2.73711491	1.88791001	0.06218200
H	2.26273394	-3.03868198	0.04604500

CPE Structure B

C	3.77411699	0.69551498	-0.05031200
C	2.57151794	1.39279497	-0.09986400
C	1.34940696	0.71217000	-0.04962400
C	1.34944606	-0.71223402	0.04914400
C	2.57163095	-1.39276695	0.09955700
C	3.77418303	-0.69537300	0.05080100
H	4.71197414	1.23764300	-0.08910000
H	4.71206808	-1.23735404	0.09067400
C	0.08484500	-1.36740100	0.07829600
C	-1.08127201	-1.67931902	0.06266200
C	-2.52417397	-1.87203002	-0.03097600
H	-2.92746997	-2.19185090	0.93777698
H	-2.74265003	-2.68427205	-0.73380500
C	-3.25835395	-0.58933997	-0.50381601
H	-2.81280804	-0.27307001	-1.45130503
H	-4.29147911	-0.87242001	-0.72707403
C	-3.25844789	0.58937103	0.50390100
H	-4.29160595	0.87244600	0.72701001
H	-2.81304002	0.27308801	1.45145094
C	-2.52420306	1.87206101	0.03123200
H	-2.74248004	2.68416500	0.73429000
H	-2.92759800	2.19217205	-0.93738699
C	-1.08136201	1.67911994	-0.06269300
C	0.08478000	1.36733902	-0.07863500
H	2.56625891	-2.47347212	0.17564100
H	2.56613994	2.47349000	-0.17621601

CPE Structure C

C	3.82500792	-0.70168900	0.00720700
C	2.63895106	-1.40252304	0.00978800
C	1.37719297	-0.76981097	-0.00145200
C	1.37721300	0.76985598	0.00156400
C	2.63903189	1.40251696	-0.00983400
C	3.82504106	0.70164198	-0.00730000
H	4.76430702	-1.24239695	0.01501500
H	4.76432610	1.24238300	-0.01514200
C	0.13814300	1.34830403	0.01167300
C	-1.05249000	0.78883302	-0.01145900
C	-2.39476490	1.49810803	-0.12410400
H	-2.53051901	1.77910304	-1.17591596
H	-2.32237196	2.43126512	0.43681699
C	-3.60682201	0.68768698	0.33178699
H	-3.59107590	0.56931198	1.42178404
H	-4.51834583	1.24071503	0.08619100
C	-3.60680294	-0.68767601	-0.33189201
H	-4.51836395	-1.24068105	-0.08638200
H	-3.59095597	-0.56929803	-1.42188704
C	-2.39479399	-1.49811399	0.12410100
H	-2.32234597	-2.43125892	-0.43683499
H	-2.53062510	-1.77912295	1.17589700
C	-1.05250299	-0.78884298	0.01157600
C	0.13813500	-1.34830296	-0.01157300
H	2.64642596	-2.48455691	0.02204700
H	2.64631391	2.48459792	-0.02208000

1,4-MeO-CPE Structure A'

C	3.06901097	-0.84815001	0.12541001
C	1.89825106	-1.58556402	-0.00464900
C	0.65273100	-0.91749698	-0.05457700
C	0.60769898	0.49223399	0.02169100
C	1.81397700	1.22073996	0.15886299
C	3.02844095	0.54619598	0.20941800
H	4.02986193	-1.34277403	0.16324200
H	3.95628309	1.09071398	0.31758299
C	-0.61853302	1.25951004	-0.11208700
C	-1.95261800	1.45127404	0.10555200
C	-3.24628496	1.08752406	0.75753099
H	-4.04863024	1.54678202	0.16897300
H	-3.24993110	1.62763596	1.71415401
C	-3.56204009	-0.39432001	1.01347399
H	-2.71950507	-0.84841502	1.54117405
H	-4.41359806	-0.43699899	1.69896400
C	-3.88497591	-1.20650196	-0.26411599
H	-4.94797802	-1.46334398	-0.29607901
H	-3.68346000	-0.59973103	-1.15192199
C	-3.05143404	-2.50415897	-0.38392201
H	-3.29660010	-3.02490807	-1.31566000
H	-3.29916692	-3.19371009	0.43239200
C	-1.63709605	-2.16007090	-0.33412299
C	-0.55209202	-1.64872897	-0.20943600
C	-1.22535098	2.48038006	-0.55782700
O	-1.16094601	3.56753111	-1.08350301
O	1.67556000	2.56701803	0.25824699
O	1.84180105	-2.94280410	-0.09680200
C	2.83411193	3.38660192	0.20817301
C	3.05774689	-3.67399096	-0.06391700
H	3.71330905	-3.40303493	-0.89947200
H	2.77484798	-4.72124004	-0.15467300
H	3.59232807	-3.52480888	0.88129002
H	3.41524100	3.19973397	-0.70136797
H	2.46559000	4.41023922	0.19736700
H	3.47081804	3.23702097	1.08775103

1,4-MeO-CPE Structure B'

C	-2.96846104	0.69874299	0.01721800
C	-1.77344501	1.40921104	0.03343000
C	-0.54843402	0.70928502	0.01536700
C	-0.54787201	-0.70899099	-0.01515000
C	-1.77234399	-1.40983796	-0.03326900
C	-2.96790910	-0.70033002	-0.01680100
H	-3.91792393	1.21598804	0.02976500
H	-3.91696405	-1.21832800	-0.02900700
C	0.71256697	-1.36715901	-0.01329500
C	1.87858200	-1.67663205	0.02017000
C	3.32126808	-1.86566603	0.11967700
H	3.72218490	-2.23154497	-0.83387500
H	3.54081106	-2.64466596	0.85913497
C	4.05999804	-0.56449097	0.52971900
H	3.61809206	-0.20313700	1.46274304
H	5.09358597	-0.83875102	0.76254100
C	4.05855989	0.56615102	-0.53145200
H	5.09149599	0.84084100	-0.76664698
H	3.61461210	0.20461901	-1.46343398
C	3.32016110	1.86702096	-0.11975200
H	3.53661203	2.64577699	-0.86037302
H	3.72385597	2.23369408	0.83231002
C	1.87794399	1.67677796	-0.01624300
C	0.71153700	1.36836100	0.01335300
O	-1.67411101	-2.76929498	-0.06472300
O	-1.67627501	2.76864791	0.06476400
C	-2.87022090	-3.53089094	-0.08197800
C	-2.87294888	3.52941108	0.08082800
H	-3.47550011	3.35309601	-0.81793898
H	-2.56059694	4.57216597	0.10428100
H	-3.47680807	3.31342101	0.97000903
H	-3.47385311	-3.31456804	-0.97123098
H	-2.55711889	-4.57340813	-0.10620100
H	-3.47329092	-3.35580111	0.81668001

1,4-MeO-CPE Structure C'

C	-2.99270701	-0.70424199	0.00098100
C	-1.81385601	-1.41917002	0.00737900
C	-0.55510199	-0.75642800	0.01572500
C	-0.55507499	0.75638002	-0.01596300
C	-1.81379402	1.41917598	-0.00744800
C	-2.99267411	0.70434397	-0.00081400
H	-3.94465899	-1.21614802	-0.00244800
H	-3.94459891	1.21629298	0.00287300
C	0.67902499	1.34759605	-0.04190000
C	1.86982298	0.79396600	0.00397600
C	3.21302700	1.49648798	0.13564600
H	3.34365797	1.76312804	1.19196200
H	3.14453697	2.43698001	-0.41318399
C	4.42647314	0.69094598	-0.32487100
H	4.41295624	0.58341002	-1.41605902
H	5.33740187	1.24188995	-0.07173500
C	4.42642784	-0.69105297	0.32495299
H	5.33736515	-1.24201703	0.07188700
H	4.41281509	-0.58352798	1.41614103
C	3.21299195	-1.49654901	-0.13566400
H	3.14451098	-2.43710303	0.41306400
H	3.34361911	-1.76307404	-1.19200897
C	1.86979496	-0.79403400	-0.00388400
C	0.67898601	-1.34764695	0.04181900
O	-1.73303604	2.78316092	0.00083800
O	-1.73320699	-2.78313088	-0.00085100
C	-2.94268489	3.52394009	0.01120500
C	-2.94289494	-3.52380490	-0.01121400
H	-2.64602709	4.57160187	0.01402100
H	-3.54830289	3.32236409	-0.88020003
H	-3.53742409	3.31425595	0.90806198
H	-3.53755593	-3.31420994	-0.90813398
H	-3.54857111	-3.32201195	0.88012201
H	-2.64635205	-4.57150078	-0.01381100

1,4-O=CPE Structure A'

C	3.53893304	-0.99513203	0.13256501
C	2.27832699	-1.74427700	-0.07521600
C	0.99940401	-0.95555300	-0.10211800
C	1.01218605	0.40511999	0.05785700
C	2.29940200	1.13063896	0.29180801
C	3.54808402	0.33059001	0.30640301
H	4.44107723	-1.59633505	0.14176001
H	4.45802402	0.89610898	0.47256699
C	-0.15864600	1.24296904	-0.08058200
C	-1.47474694	1.50517201	0.14541601
C	-2.74691510	1.19888496	0.86495900
H	-3.55517292	1.72225595	0.34366399
H	-2.66328406	1.69075596	1.84308004
C	-3.11487889	-0.28027499	1.07048094
H	-2.27402496	-0.78975302	1.54870403
H	-3.94082689	-0.31830201	1.78600395
C	-3.51607990	-1.02269602	-0.22804300
H	-4.59085798	-1.22378194	-0.23732799
H	-3.30997801	-0.39252600	-1.09823501
C	-2.75892401	-2.35843992	-0.42599100
H	-3.05568600	-2.82789397	-1.36960804
H	-3.01479506	-3.07001805	0.36826199
C	-1.32816601	-2.09736395	-0.40657300
C	-0.21236600	-1.65072703	-0.29221901
C	-0.73199397	2.45773411	-0.61095297
O	-0.63689601	3.48368907	-1.23177004
O	2.32370710	2.33250403	0.47970501
O	2.27616501	-2.95249009	-0.20863700

1,4-O=CPE Structure B'

C	-3.52069497	0.66815400	0.03043500
C	-2.26057696	1.45246303	0.06518700
C	-0.97877002	0.68818599	0.02825700
C	-0.97888100	-0.68835700	-0.02855600
C	-2.26082110	-1.45242095	-0.06537700
C	-3.52079797	-0.66774702	-0.03040500
H	-4.43205214	1.25492895	0.05731100
H	-4.43227816	-1.25436294	-0.05707800
C	0.25944701	-1.37051797	-0.04283300
C	1.42944801	-1.67114997	-0.01028800
C	2.86933804	-1.86672902	0.08561800
H	3.26096010	-2.22203207	-0.87534899
H	3.08081102	-2.66119504	0.81033802
C	3.61813998	-0.57756901	0.51647902
H	3.19038391	-0.23545800	1.46322596
H	4.65108109	-0.86555398	0.73198003
C	3.61686802	0.57766497	-0.51832199
H	4.64929295	0.86537200	-0.73661000
H	3.18635988	0.23573500	-1.46387994
C	2.86939192	1.86670399	-0.08493800
H	3.07906508	2.66189790	-0.80934900
H	3.26337194	2.22108197	0.87542301
C	1.42965496	1.67102504	0.01361100
C	0.25963199	1.37011194	0.04203900
O	-2.28060699	-2.66803002	-0.12146600
O	-2.28005004	2.66811395	0.12128500

1,4-O=CPE Structure C'

C	-3.54911804	-0.66783899	-0.01090500
C	-2.30806994	-1.47407901	-0.02386700
C	-0.99157602	-0.75505602	-0.01365900
C	-0.99157000	0.75504899	0.01375600
C	-2.30805993	1.47407901	0.02392500
C	-3.54911399	0.66786200	0.01044700
H	-4.46467400	-1.24863005	-0.02017300
H	-4.46466684	1.24865997	0.01943600
C	0.21497799	1.33793795	0.02533400
C	1.42383206	0.75139600	0.00908900
C	2.75165105	1.49828506	0.03632800
H	2.88845897	1.87935495	1.05551398
H	2.64852595	2.37581205	-0.60464698
C	3.96825790	0.66872400	-0.36891699
H	3.95547199	0.49097100	-1.45056498
H	4.87734222	1.23656702	-0.15247500
C	3.96822810	-0.66872400	0.36884499
H	4.87732220	-1.23660600	0.15253800
H	3.95538402	-0.49092701	1.45049095
C	2.75164795	-1.49829400	-0.03639800
H	2.64855790	-2.37583208	0.60457200
H	2.88841605	-1.87931705	-1.05560696
C	1.42383003	-0.75140899	-0.00910500
C	0.21497400	-1.33793795	-0.02529200
O	-2.37058496	2.68734288	0.04307400
O	-2.37060094	-2.68734598	-0.04264500

1,4-CN-CPE Structure A'

C	3.31260991	-0.93188900	0.16572900
C	2.11097908	-1.62392902	-0.01625400
C	0.88119900	-0.92870200	-0.08811000
C	0.87792599	0.48464900	0.01050500
C	2.10565400	1.15854800	0.19265001
C	3.30940509	0.44858801	0.27730399
H	4.24150276	-1.48445594	0.22054100
H	4.23433590	0.98946100	0.42826399
C	-0.34093800	1.26504099	-0.15080599
C	-1.65807199	1.48190606	0.11454500
C	-2.90228200	1.17556703	0.88074702
H	-3.72888494	1.68805099	0.37830299
H	-2.78681803	1.68328905	1.84757197
C	-3.26526189	-0.29936999	1.12529600
H	-2.42290592	-0.79668802	1.61289299
H	-4.08708715	-0.31636000	1.84609604
C	-3.67657304	-1.07382905	-0.15170100
H	-4.74208689	-1.31687903	-0.12546600
H	-3.52311301	-0.44788799	-1.03562999
C	-2.87729406	-2.38394594	-0.35569599
H	-3.16966891	-2.86631298	-1.29382205
H	-3.09777188	-3.09927297	0.44525301
C	-1.45574701	-2.07517600	-0.35470399
C	-0.34767199	-1.61038995	-0.25481799
C	-0.99282998	2.40984607	-0.74088198
O	-0.97446603	3.38987207	-1.43782496
C	2.14136004	2.58174491	0.33602300
C	2.13395905	-3.04935908	-0.12159000
N	2.21069002	3.72502804	0.48245099
N	2.18069696	-4.20041895	-0.20114100

1,4-CN-CPE Structure B'

C	-3.26292610	0.69153303	0.01704500
C	-2.05205202	1.39828706	0.03328600
C	-0.82043499	0.71331000	0.01521800
C	-0.82031000	-0.71331197	-0.01527800
C	-2.05180812	-1.39864397	-0.03348600
C	-3.26282692	-0.69215202	-0.01729200
H	-4.19635582	1.23885405	0.03050600
H	-4.19616985	-1.23960102	-0.03084800
C	0.43919501	-1.36473405	-0.01563600
C	1.60906994	-1.65973794	0.01562100
C	3.04881811	-1.86177301	0.10111300
H	3.42991996	-2.22261095	-0.86170399
H	3.26082802	-2.65367103	0.82805997
C	3.80795503	-0.57442099	0.51925498
H	3.39302897	-0.22445500	1.46884596
H	4.84056807	-0.86922199	0.72598201
C	3.80643702	0.57512099	-0.52158499
H	4.83846378	0.86973900	-0.73138601
H	3.38851905	0.22536200	-1.46993995
C	3.04855204	1.86213195	-0.10028700
H	3.25867295	2.65522289	-0.82643902
H	3.43204498	2.22151804	0.86214697
C	1.60891998	1.66002500	-0.01225400
C	0.43895200	1.36494005	0.01544200
C	-2.07621503	-2.82770300	-0.06656400
C	-2.07695699	2.82736707	0.06621800
N	-2.12485790	-3.98130202	-0.09312600
N	-2.12596011	3.98093510	0.09310800

1,4-CN-CPE Structure C'

C	3.28604698	-0.69639897	0.00339400
C	2.09148192	-1.40704405	0.00249500
C	0.82720101	-0.76121098	-0.00482800
C	0.82721198	0.76123202	0.00526500
C	2.09152102	1.40697300	-0.00241600
C	3.28606701	0.69633597	-0.00331000
H	4.22226381	-1.23901904	0.00793400
H	4.22229195	1.23893404	-0.00788400
C	-0.40782401	1.34887004	0.01746000
C	-1.59799695	0.79975098	-0.00467800
C	-2.94384789	1.50169694	-0.09963700
H	-3.07483697	1.80071199	-1.14659500
H	-2.86696196	2.42433095	0.47747999
C	-4.15333223	0.68181401	0.34313399
H	-4.13949108	0.54796201	1.43096900
H	-5.06315517	1.23943496	0.10448700
C	-4.15327406	-0.68175101	-0.34337899
H	-5.06315422	-1.23935997	-0.10491800
H	-4.13921785	-0.54789001	-1.43120897
C	-2.94388509	-1.50165796	0.09961200
H	-2.86688900	-2.42425799	-0.47754699
H	-3.07509708	-1.80074096	1.14652205
C	-1.59800696	-0.79971999	0.00499300
C	-0.40783799	-1.34885395	-0.01717500
C	2.13712001	2.83634496	-0.00990300
C	2.13704395	-2.83636498	0.00964300
N	2.21279907	3.98892593	-0.01608300
N	2.21264601	-3.98895502	0.01561700
